# Transgenic mouse models for investigating human *DUX4* expression during development and its roles in FSHD pathophysiology

**DOI:** 10.1242/dmm.052637

**Published:** 2026-05-18

**Authors:** Yosuke Hiramuki, Charis L. Himeda, Peter L. Jones, Takako I. Jones

**Affiliations:** Department of Pharmacology, Center for Molecular Medicine, University of Nevada, Reno School of Medicine, 1664 N Virginia St., Reno, NV 89557, USA

**Keywords:** Pericyte, Skeletal muscle, Facioscapulohumeral muscular dystrophy, Mouse model for FSHD

## Abstract

Facioscapulohumeral muscular dystrophy (FSHD) is an autosomal dominant myopathy caused by aberrant expression of the double homeobox 4 (*DUX4*) retrogene, affecting skeletal muscles primarily in the face, shoulder and limbs. In healthy individuals, *DUX4* is expressed in early development and subsequently silenced in most somatic tissues. The spatiotemporal pattern of DUX4 misexpression beyond the cleavage stage in FSHD is poorly understood because *DUX4* is not well conserved beyond primates. Here, we generated Cre reporter mouse lines with human *DUX4* regulatory elements to investigate the cell lineages derived from *DUX4*-expressing cells in embryos and adults. Intriguingly, we found that *DUX4*-expressing cell lineages are present in embryonic forelimb, hindlimb and face. In adults, the reporter was expressed strongly in testis and, to a lesser extent, in other tissues, including weak sporadic expression in skeletal muscles, which is reminiscent of mosaic DUX4 expression in FSHD. Within skeletal muscles, DUX4 lineage cells include pericytes, interstitial cells that contribute to muscle regeneration and repair. Overall, this study introduces a new research tool for the field and provides new insight into potential developmental mechanisms underlying FSHD pathophysiology.

## Teaser

**Summary:** Cre-reporter mouse lines expressing human *DUX4* regulatory elements are discovery tools for developmental processes and mechanisms underlying FSHD pathophysiology.

## INTRODUCTION

Facioscapulohumeral muscular dystrophy (FSHD) is the third-most-common autosomal dominant muscle disorder with a prevalence of 7-12 per 100,000 individuals worldwide (Deenen et al., 2014, 2025) (Orphanet, 2024). While FSHD pathology is highly variable, it typically affects muscles of the face, shoulder blades and upper arms initially, and then progresses to the abdomen and lower legs, with much of the weakness and atrophy appearing asymmetrically. Ultimately, all skeletal muscles are at risk of becoming affected (Mul, 2022; Padberg, 1982). In addition, extra-muscular disease manifestations can occur in the severe infantile form of FSHD, including high-frequency hearing loss and retinal abnormalities (Chen et al., 2020; Goselink et al., 2017; Klinge et al., 2006). Many clinical characteristics of FSHD are unusual among neuromuscular diseases and the underlying pathological mechanism(s) are not fully understood.

Both FSHD1 and FSHD2 are caused by the loss of local epigenetic repression, resulting in aberrantly increased expression of the double homeobox 4 (*DUX4*) retrogene from the chromosome 4q35 D4Z4 array (Himeda and Jones, 2019; Lemmers et al., 2010; Snider et al., 2010). *DUX4* encodes a double homeobox C (DUXC) family transcription factor that, similar to other *DUXC* genes, initiates an early zygotic gene expression program in cleavage stage embryos, after which it is silenced in healthy somatic cells (De Iaco et al., 2017; Dixit et al., 2007; Gabriëls et al., 1999; Hendrickson et al., 2017; Kowaljow et al., 2007; Leidenroth et al., 2012; Nip et al., 2023; Whiddon et al., 2017). However, in FSHD, *DUX4* is epigenetically de-repressed, leading to aberrant upregulation of its mRNA, protein and target genes in skeletal muscles. Interestingly, aberrantly increased expression of DUX4-target genes has been detected in human FSHD fetal muscle biopsies as early as 14 weeks (Ferreboeuf et al., 2014). Initial expression of DUX4 establishes an epigenetic signature at its target genes that primes them for expression upon later exposure to DUX4 (Resnick et al., 2019); this suggests that early DUX4 expression can have a developmental role in dictating later FSHD pathology. The only healthy adult tissue where DUX4 protein is known to be expressed is testis, although *DUX4* mRNA has been reported in the thymus, cultured human keratinocytes (Gannon et al., 2016) and *in-vitro*-derived osteoblasts (de la Kethulle de Ryhove et al., 2015). Overall, very little is known about the pattern of *DUX4* expression in healthy or FSHD states *in vivo*. Although all placental organisms have a *DUXC* ortholog, human *DUX4* is primate specific, rendering it difficult to study developmentally in traditional model organisms, and all FSHD animal models are engineered transgenics (Bosnakovski et al., 2017, 2022; Jones and Jones, 2018; Jones et al., 2016; Pakula et al., 2019; Wuebbles et al., 2010). Thus, any developmental role for endogenous DUX4 expression in mediating typical or atypical FSHD pathology is still unknown.

Here, we took a transgenic approach to investigate the developmental expression of *DUX4.* Previously, we have characterized two D4Z4-proximal enhancers that drive muscle-specific expression of full-length *DUX4* (*DUX4-fl*) (Himeda et al., 2014). These *DUX4* myogenic enhancer regions 1 and 2 (DME1 and DME2, respectively), which are not present in the mouse genome are, respectively, ∼4 kb and ∼15 kb centromeric to the 4q35 D4Z4 array, and contain transcription factor binding motifs associated with early development and skeletal muscle gene expression (Himeda et al., 2014). Interestingly, one FSHD patient family has been identified whose members show a natural deletion of the region encompassing these DMEs on the permissive allele (Deak et al., 2007; Lemmers et al., 2022; Nguyen et al., 2019). However, due to the trans-acting nature of enhancers, it is certainly possible that DME sequences on the other 4q allele or either 10q allele can interact with the de-repressed *DUX4* promoter and help to drive *DUX4-fl* expression. In addition to the DMEs, others have identified putative regulatory elements within the D4Z4 repeat itself (Dmitriev et al., 2011; Gabriëls et al., 1999; Ottaviani et al., 2010). Using these findings, we assembled a reporter transgene containing *cis* human *DUX4* regulatory elements including the DME regions. We replaced the *DUX4*-coding sequence with Cre recombinase-EGFP to eliminate the cytotoxic effect of DUX4 and allow cell-lineage tracing during embryogenesis and in adult tissues with the desired floxed reporter lines. Using the sensitive floxed *lacZ* reporter mouse, we observed positive cell lineages in the face and limbs of embryos, and in testis, heart and skeletal muscles of adults, and identified blood-vessel-associated pericytes as a previously unreported DUX4-expressing cell lineage.

## RESULTS

### Generation of pJ2-Cre:EGFP mice

**Fig. 1.**
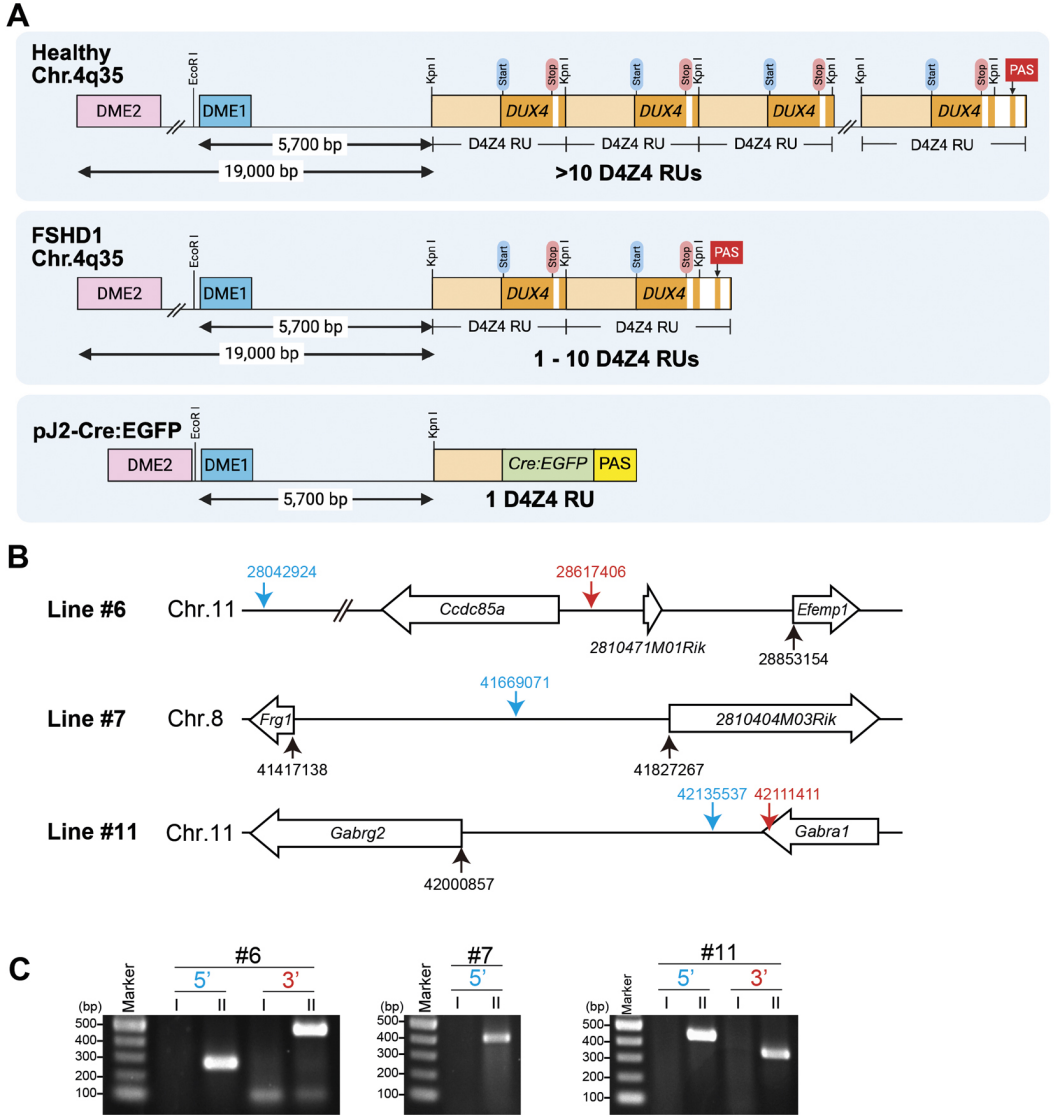
**Generation of pJ2-Cre:EGFP mice.** (A) Diagram of the human chromosome 4q35 *D4Z4/DUX4* locus in healthy and FSHD1 individuals, and in the pJ2-Cre:EGFP construct. Each D4Z4 repeat unit (D4Z4 RU) (3303 bp) contains *DUX4* exons (dark orange) 1 and 2, with the last D4Z4 RU also containing exon 3, which includes the polyadenylation signal (PAS) that stabilizes *DUX4-fl* in the FSHD condition. Also indicated are the 1230 bp DME1 sequence (blue), 2100 bp DME2 sequence (pink) and D4Z4 sequence without *DUX4* (light orange). For pJ2-Cre:EGFP, *DUX4* is replaced by a Cre:EGFP fusion gene (green) and a β-globin PAS (yellow). (B) Diagrams showing the integration sites of the three mouse lines (#6, #7 and #11). Blue and red arrows indicate the 5′ and 3′ integration sites of transgenes, respectively. Genomic locus position was compared to the mouse mm10 sequence. (C) Agarose gels showing PCR results to validate the transgene integration sites. I and II indicate *R26^NZG/+^* and *pJ2-Cre:EGFP; R26^NZG/+^*mice, respectively, for lines #6, #7 and #11. For line #6, the 5′ site (283 bp) and 3′ site (495 bp) products are shown; for line #7, only the 5′ site (411 bp) was identified. For line #11, the 5′ (453 bp) and 3′ (365 bp) products are shown. DME1 and 2, DUX4 myogenic enhancer 1 and 2, respectively; PAS, polyadenylation signal.

We were interested in understanding the developmental expression of DUX4 in FSHD; however, due to the lack of evolutionary conserved orthologs outside of primates, the synteny of the region and genome organization of the repeat array, there were no suitable *in vivo* vertebrate developmental systems available. Although the *DUX4*-coding sequence itself is primate specific, the *DUX* family of double homeodomain protein-encoding genes as well as D4Z4-like repeats are conserved between primates and mice (Clapp et al., 2007; Leidenroth et al., 2012; Leidenroth and Hewitt, 2010), suggesting that mice have the capacity to appropriately regulate the expression of human *DUX4*. Therefore, we created a transgene consisting of the known endogenous *cis* transcriptional *DUX4*-regulatory elements to generate transgenic mice that recapitulate developmental *DUX4* expression profiles. The transgene construct pJ2-Cre:EGFP consists of the two *DUX4* myogenic enhancers (1230-bp DME1 and 2100-bp DME2) (Himeda et al., 2014), a single chromosomal tandem D4Z4 repeat unit (RU) containing the core *DUX4* promoter elements and any regulatory elements within the repeat (Dmitriev et al., 2011; Gabriëls et al., 1999; Ottaviani et al., 2010), the Cre-EGFP fusion gene in place of the *DUX4* open reading frame and a β-globin PAS (Fig. 1A). Specifically, the transgene contains the endogenous 5700-bp sequence proximal to the most-centromeric 4q35 D4Z4 RU – from the KpnI restriction site to DME1 – of human chromosome 4q35. DME2, located ∼19 kb proximal to the most-centromeric 4q35 D4Z4 RU, was placed upstream and directly adjacent to DME1.

Transgenic mice were generated by random integration of the linearized construct into B6;SJL hybrid eggs using standard protocols. The resultant progeny were backcrossed to C57BL/6J and screened for the transgene by PCR. During backcrossing, three independent lines of pJ2-Cre:EGFP mice (hereafter referred to as #6, #7 and #11) were selected for relatively normal Mendelian inheritance of the transgene, an indication of no embryonic lethality due to insertion location. To eliminate genetic background that may contribute to variable expression patterns between lines and within the litters of each line, all three lines were backcrossed to C57BL/6 ten times to establish congenic lines before analysis. Genomic mapping analysis was performed on these lines using targeted locus amplification (TLA) to identify the transgene integration sites and provide estimates on transgene copy number. The mouse reference genome assembly GRCm38 (mm10) (https://www.ncbi.nlm.nih.gov/datasets/genome/GCF_000001635.20/) was used as the reference sequence for alignment between transgene and host genome (Fig. 1B). The integration site for line pJ2-Cre:EGFP (#6) was at chromosome 11: 28042924 - 28617406. According to the reference sequence, the integration event affects the coiled-coil domain-containing 85A (*Ccdc85a*) and *2810471M01Rik* gene regions. A complex integration has occurred here with different genomic rearrangements including transgene inversion, fusion and deletion. The copy number was estimated to be three to six copies. The pJ2-Cre:EGFP (#7) 5′ integration site was determined to be chromosome 8 (8:41669071), which is near no annotated genes; however, the 3′ integration site could not be identified, suggesting it is within a repetitive or low-complexity region; such regions are less efficiently sequenced and show low to no sequence coverage. The copy number was estimated to be 24-33 copies. The pJ2-Cre:EGFP (#11) transgene was integrated into chromosome 11 (11:42111411-42135537). The 24-kb genomic sequence between the two identified breakpoints was duplicated and present at both ends of the integrated sequence. The integration event and genomic duplication included exons 9 and 10 of the gamma-aminobutyric acid [GABA] type A receptor subunit alpha1 (*Gabra1*) gene. The copy number was estimated to be 28-78 copies. To confirm these mapping analyses, we performed genomic PCR with primers between the transgene and host genome in each of the three lines and detected a PCR product of the expected size in all cases (Fig. 1C).

### *DUX4*-expressing cell lineages are present in the forelimb, hindlimb and face during development

To visualize cell lineages where the enhancers of *DUX4* and its promoter have been active, we used *R26^NZG^* reporter mice (Yamamoto et al., 2009), which express a nuclear localized β-galactosidase (β-gal) from the *lacZ* transgene under the control of the ubiquitous CAG promoter in response to Cre recombinase expression (Fig. S1A). Thus, in double transgenic mice, any cell in which the *DUX4* regulatory elements were active would express Cre, leading to stable recombination of the reporter and expression of β-gal in the subsequent cell lineages, which is detected by X-gal staining at high sensitivity. Since some enhancers are active during oogenesis and Cre protein persists post meiotically in the oocyte, we first evaluated whether pJ2-Cre:EGFP has maternally inherited activity. We found that embryos produced from female *pJ2-Cre:EGFP/+* mice crossed with male *R26^NZG/NZG^* reporter mice showed a recombined transgene in the amniotic sac and umbilical cord, and expressed ubiquitous β-gal regardless of inheritance of the pJ2-Cre:EGFP transgene (Fig. S1B,C). This indicates that the *DUX4* regulatory elements are active in the female germline. Fortunately, embryos produced from female *R26^NZG^* mice crossed with male *pJ2-Cre:EGFP/+* mice did not show Cre activity in the umbilical cord or amniotic sac (Fig. S1D), indicating no paternal inheritance of Cre activity. Thus, all future litters in this study were generated using male *pJ2-Cre:EGFP* mice crossed with female *R26^NZG^* mice.

**Fig. 2.**
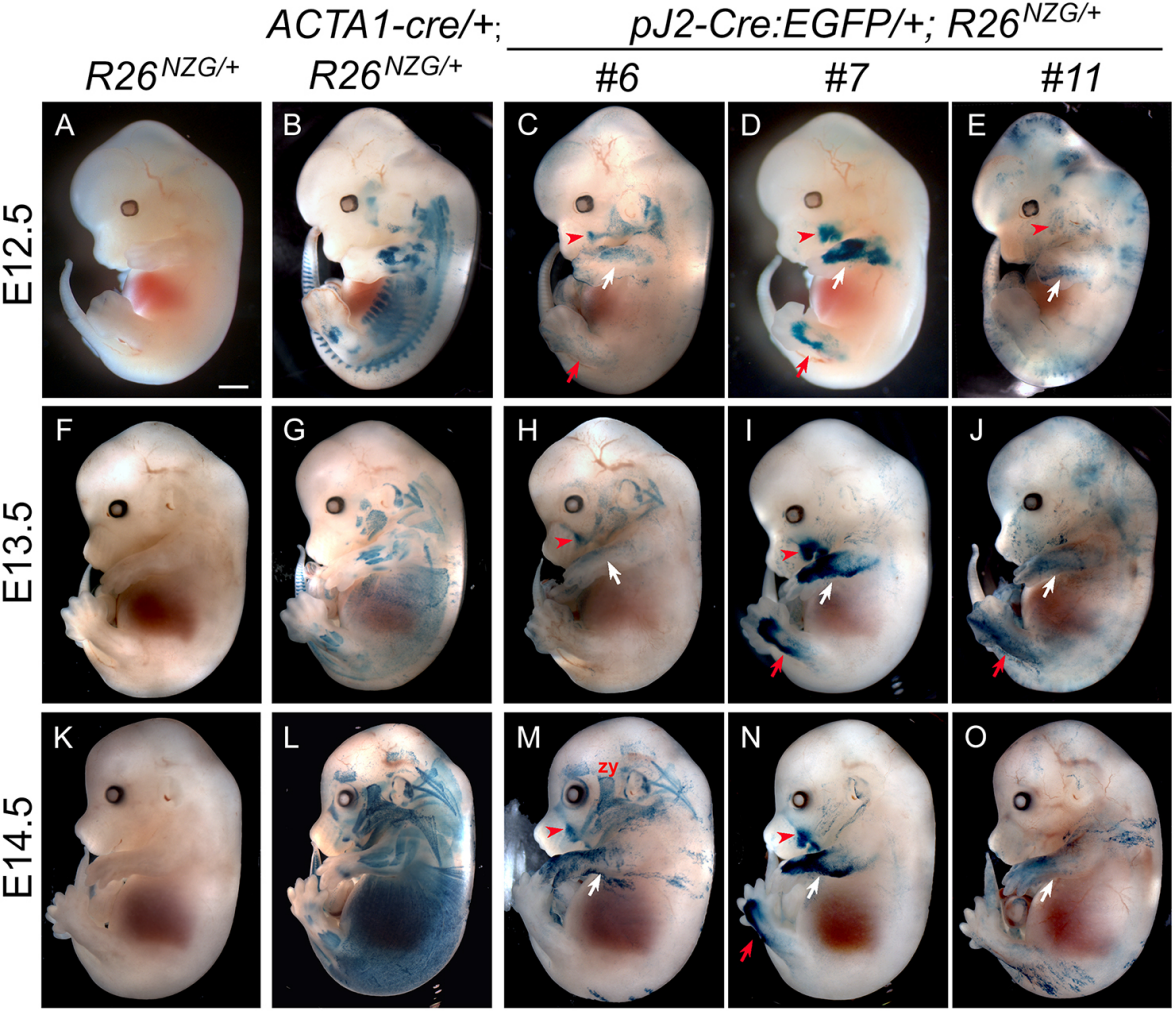
**Activity of *DUX4* regulatory elements in limb and face during murine embryonic development.** (A-O) X-gal staining pattern in embryos; *pJ2-Cre:EGFP/+; R26*^*NZG/+*^ double transgenic embryos at E12.5 (C-E), E13.5 (H-J), and E14.5 (M-O). In all three independent insertion lines of the transgene (#6, #7 and #11), *DUX4* regulatory elements are active in forelimb (white arrows), hindlimb (red arrows), and face where the zygomaticus muscle (zy) connects at the corner of the lip (red arrowheads). X-gal staining pattern of embryonic skeletal muscles in *ACTA1-cre/+; R26*^*NZG/+*^ double transgenic embryos are shown at each embryonic stage as an example of pan skeletal muscle staining (B,G,L) along with embryos containing only the reporter transgene (A,F,K) as a negative control. Scale bar: 1 mm.

During development, myogenesis occurs in two stages: primary myogenesis in which PAX3-positive progenitors arise from the dermomyotome to form multinucleated primary myofibers (embryonic stage E10-E12), and secondary myogenesis in which PAX7-positive progenitors form secondary fibers by using primary fibers as a scaffold, thus contributing to the growth of fetal muscle (E14.5-E17.5) (Chal and Pourquie, 2017). To determine if the *DUX4* regulatory elements are active during embryonic myogenesis, *pJ2-Cre:EGFP/+; R26^NZG/+^* double transgenic embryos were generated from the three independent-insertion lines #6, #7 and #11, and reporter expression was analyzed at various stages. Intriguingly, X-gal-positive cells were commonly detected close to the dermis in both limbs and at the corner of the mouth in all three mouse lines during E12.5 to E14.5, although the pattern, intensity and timing of β-gal expression were variable (Fig. 2; Figs S2, S3). Since FSHD is a skeletal muscle disease and the *DUX4* transgene contains two myogenic enhancers, it was expected that expression would be found in skeletal muscle lineages. To determine the X-gal staining patterns for skeletal muscle lineages, *ACTA1-Cre/+; R26^NZG/+^* embryos were analyzed (Fig. 2; Figs S2, S3). Surprisingly, all lines showed staining minimally overlapping with that of *ACTA1-Cre/+; R26^NZG/+^* embryos, except line #6, which showed staining in facial expression muscles located close to the surface (Lescroart et al., 2010) (Fig. 2; Fig. S3C-E). We identified the following X-gal-positive facial expression muscles: auricularis (au; connects the ear to the skull), buccinator (bu; used for chewing, sucking and blowing), frontalis (fr; raises the eyebrow), orbitalis oculi (oo; closes the eyelid), quadratus labii (qua; moves the upper lip, used for smiling), and zygomaticus (zy; used for smiling). The facial expression muscles originate from mesodermal progenitor cells in the second branchial arch (BA2). We confirmed that line #6 *pJ2-Cre:EGFP/+; R26^NZG/+^* embryos showed X-gal-positive cells in BA2 at E10.5 (Fig. S3A), indicating that *DUX4* regulatory elements in line #6 are active in the branchiomeric muscle lineage that gives rise to facial expression muscles. Line #11 showed similar but less consistent X-gal staining in the face (Fig. 2E; Fig. S2D), with more widespread staining in head and ventral trunk close to the surface (Fig. 2E,J,O). It is possible that reporter expression in line #6 is influenced by regulation of the neighboring *Efemp1* gene, which is expressed in BA1, BA2 and BA3 at the same stage (Ehlermann et al., 2003), thus resulting in a more extensive pattern of facial expression than the other two insertion lines.

**Fig. 3.**
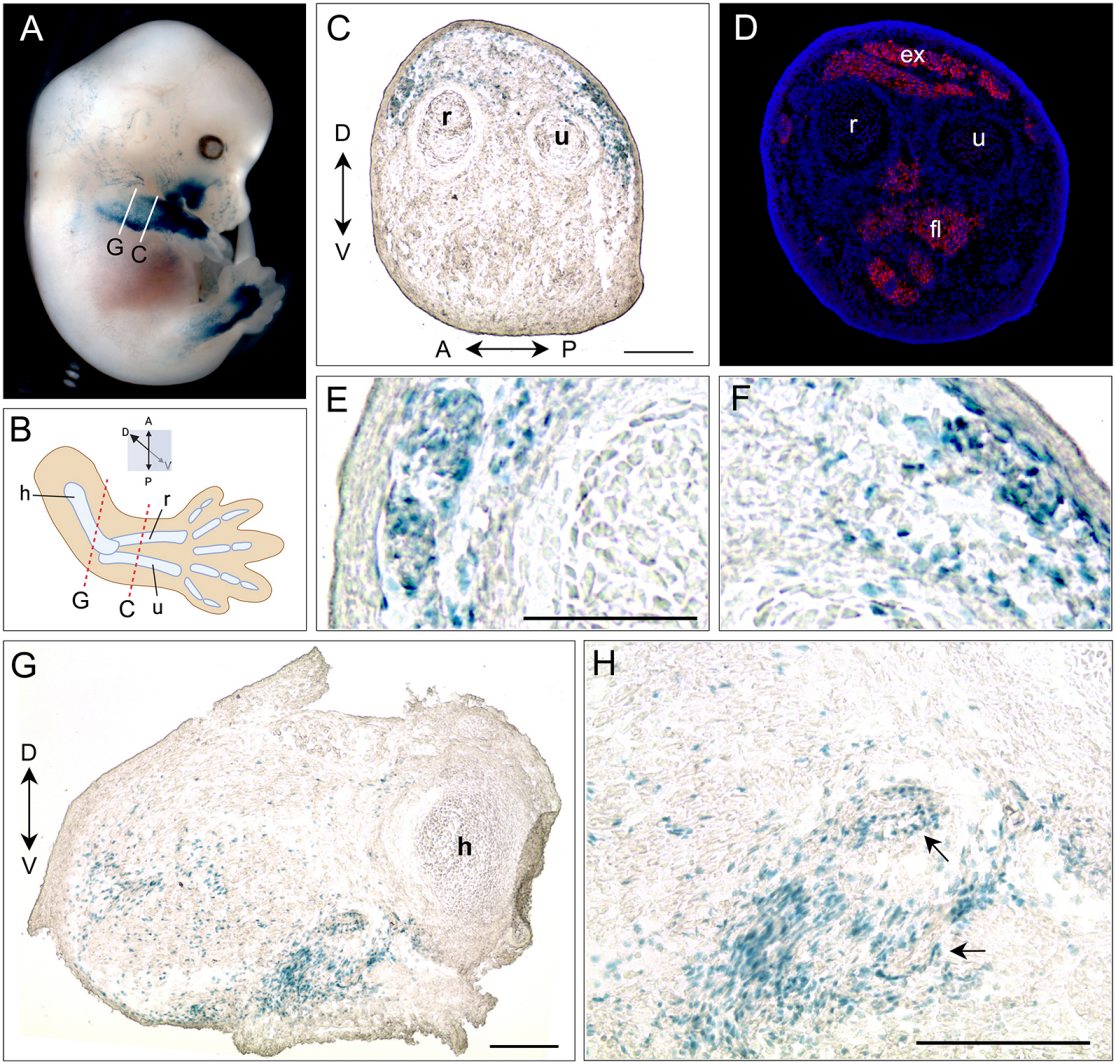
**pJ2-Cre:EGFP #7/+; *R26^NZG/+^* embryos show very localized X-gal-positive cells in forelimb, hindlimb, and the side of the mouth.** (A) Right forelimb of X-gal stained E13.5 embryo was sectioned at the places indicated (white lines labeled C and G, which are the sections shown in panels C and G). (B) Simplified illustration of forelimb with the radius (r), ulna (u) and humerus (h) bones, and anterior (A), posterior (P), dorsal (D) and ventral (V) directions. Sectioned planes are indicated by red dashed lines. (C,E,F) X-gal staining in subepidermal mesenchymal cells at dorsal part of lower forelimb. (D) The serial section of C was stained with antibodies to myosin heavy chain 1 (MF20, red); nuclei were stained with DAPI (blue). The extensor (ex) and flexor (fl) muscles are indicated. (G,H) X-gal signal in ventral mesenchyme of upper forelimb, and in cells surrounding vascular structures (arrows). Scale bars: 200 µm.

Within each line, X-gal staining among littermates was more variable compared to that of *ACTA1-Cre/+; R26^NZG/+^* embryos, except for line #7 *pJ2-Cre:EGFP/+; R26^NZG/+^*. Although we cannot rule out the possibility of integration site effects, line #7 displayed the most consistent staining pattern in forelimbs, hindlimbs and face, both throughout embryogenesis and within litters (Fig. 2D,I,N; Fig. S2C; Table S1). This strong, but sporadic staining pattern is consistent with the sporadic bursts of endogenous DUX4 expression seen in embryonic stem cells and in FSHD muscle cells. To further evaluate the identity and location of X-gal-positive cell lineages, we analyzed tissue sections from the forelimb of line #7 *pJ2-Cre:EGFP/+; R26^NZG/+^* E13.5 embryos by X-gal staining and immunostaining for myosin heavy chain 1 (MYH1) (Fig. 3). In the lower forelimb, X-gal-positive cells were observed in the dorsal mesenchyme adjacent to extensor muscles positive for MYH1 (Fig. 3C,D) just below the ectoderm (Fig. 3E,F). In the upper forelimb, X-gal staining was observed in the ventral mesenchyme next to the humerus and in the cells surrounding vascular structures (Fig. 3G,H).

Overall, in the three insertion lines, cell lineages in which the *DUX4* regulatory elements are active were observed in the face and the developing mesenchyme of the dorsal−anterior lower forelimb and lower hindlimb, but there was minimal overlap with embryonic myofibers.

### Adult tissues contain *DUX4*-expressing cell lineages

**Fig. 4.**
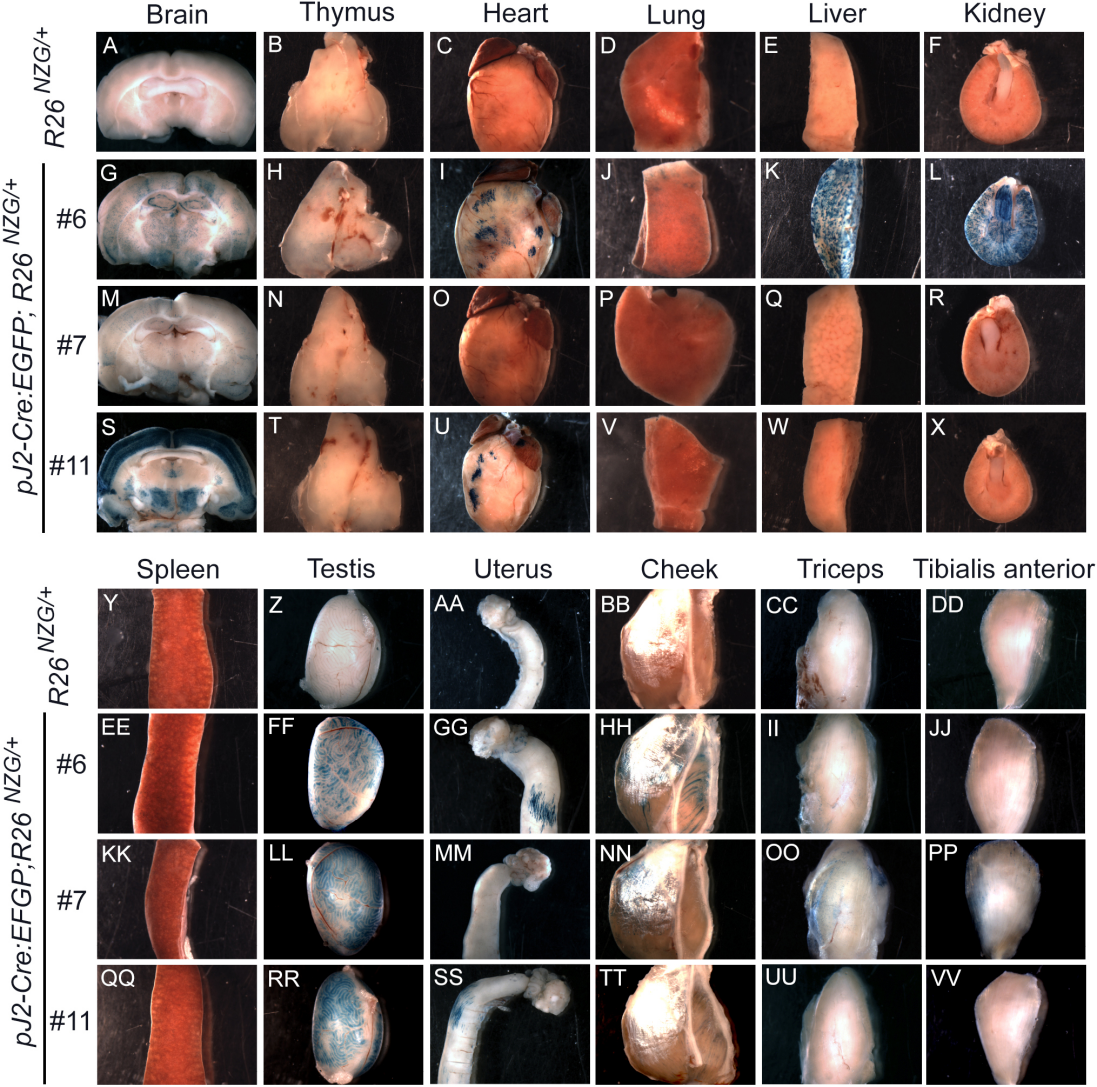
**Activity of *DUX4* regulatory elements in adult murine tissues.** (A-VV) Images showing X-gal staining for β-gal activity in the brain, thymus, heart, lung, liver, kidney, spleen, testis, uterus and skeletal muscles (cheek, triceps and tibialis anterior) of >8-week-old *R26^NZG/+^* mice and *pJ2-Cre:EGFP/+; R26^NZG/+^* mice (lines #6 , #7 and #11) as indicated.

**Fig. 5.**
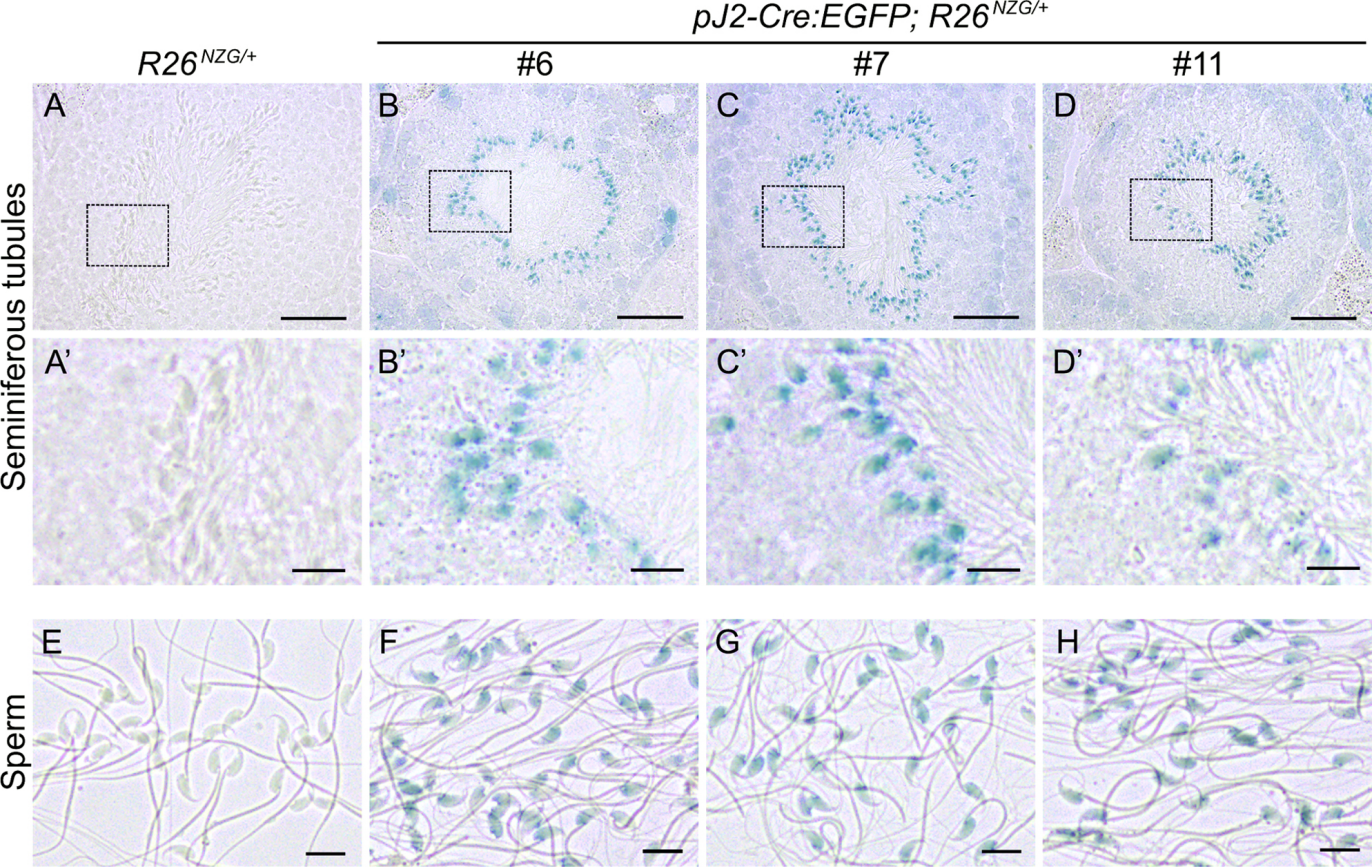
**Activity of *DUX4* regulatory elements in male germ cells.** (A-D′) X-gal staining in testis cross-sections of *R26*^*NZG/+*^ mice and *pJ2-Cre:EGFP/+; R26*^*NZG/+*^ mice (lines #6, #7 and #11) at >8 weeks of age. Boxed areas in A-D are shown magnified below (A′-D′). Scale bars: 50 μm (A-D) and 10 μm (A′-D′). (E-H) Mature sperm from ∼8-week-old mice. Scale bars: 10 µm.

We next investigated the activity of *DUX4* regulatory elements in adult tissues using the same *R26^NGZ^* reporter mice. Since most X-gal-positive lineages in *pJ2-Cre:EGFP/+; R26^NGZ/+^* embryos were in the mesenchyme outside of embryonic myofibers, we analyzed non-muscle tissues (brain, thymus, heart, lung, liver, kidney, spleen, testis and uterus) in addition to skeletal muscles [cheek, triceps and tibialis anterior (TA)] of adult mice (Fig. 4). Among these, testis was strongly X-gal-positive in all three independent-insertion lines (Fig. 4), confirming that the transgene constructs recapitulated what little is known about *DUX4* expression in adult tissues. X-gal staining of a testis cross-section showed X-gal-positive elongating spermatids at the late stage of spermatogenesis, close to the lumen in seminiferous tubules (Fig. 5A-D). In addition, mature sperm isolated from *pJ2-Cre:EGFP/+; R26^NGZ/+^* males at ∼8 weeks of age were X-gal-positive in all three lines (Fig. 5E-H). For lines #6 and #11, the X-gal signals in skeletal muscle as well as thymus, heart, lung, spleen and uterus were relatively weak (Fig. 4). Line-specific X-gal staining was observed in liver and kidney in line #6 *pJ2-Cre:EGFP; R26^NZG/+^* mice and in part of the brain in line #11 *pJ2-Cre:EGFP; R26^NZG/+^* mice. The latter was potentially a result of integration near the *Gabra1* locus, since Gabra1 functions as a receptor for the GABA neurotransmitter in the central nervous system (Gilsoul et al., 2019).

Line #7, which displayed consistent X-gal staining in the embryonic limbs and face, showed weak X-gal staining in adult muscles and strong X-gal staining in testis, without apparent staining in other non-muscle tissues, recapitulating what is known about *DUX4* expression in FSHD patients.

### Pericytes are a *DUX4*-expressing cell lineage

**Fig. 6.**
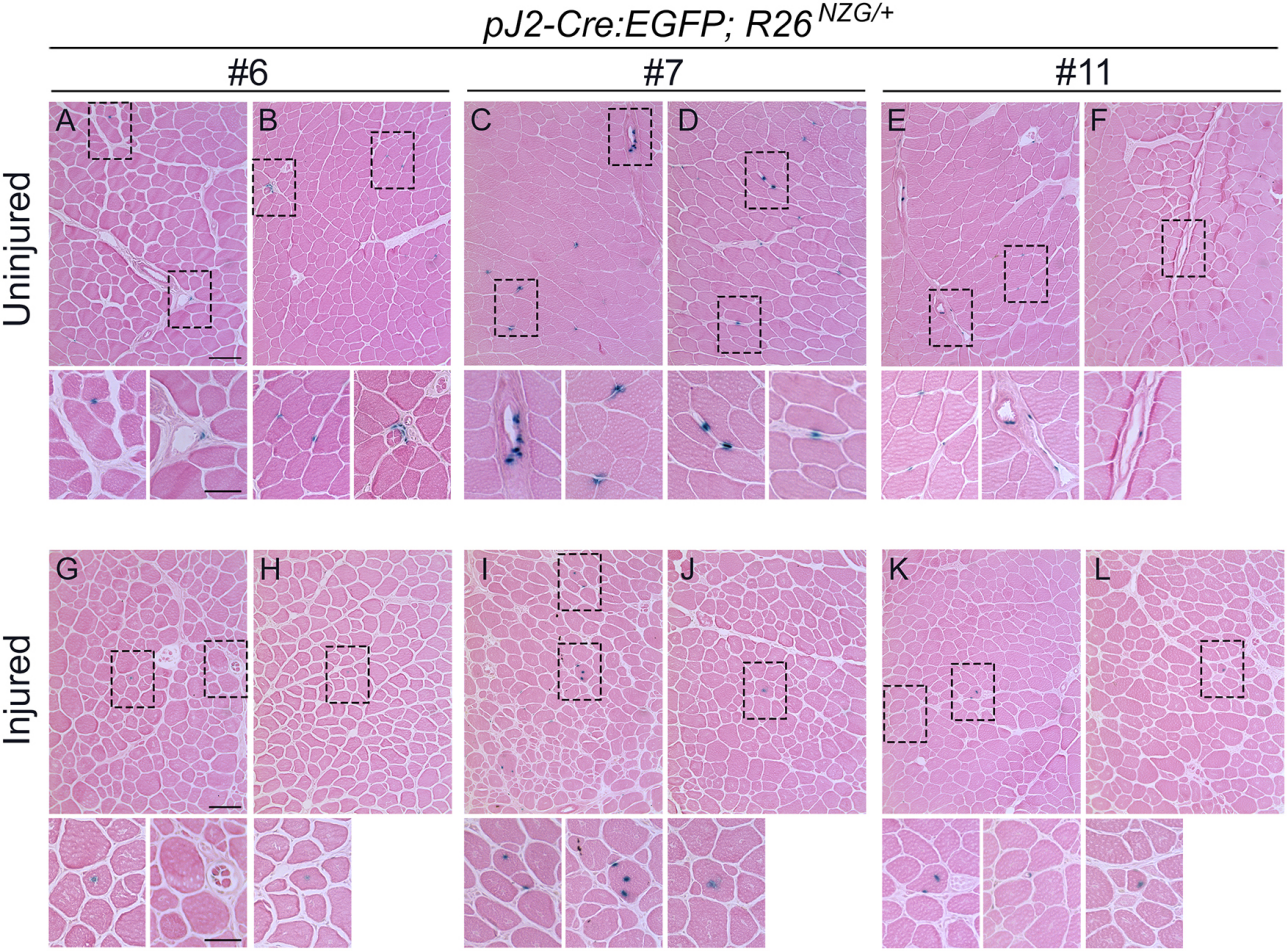
**Activity of *DUX4* regulatory elements in adult skeletal muscle.** (A-F) X-gal and Eosin staining of tibialis anterior (TA) muscle sections obtained from *pJ2-Cre:EGFP/+; R26^NZG/+^* mouse lines #6 (A,B), #7 (C,D) and #11 (E,F) at >8 weeks of age (*n*=2 animals per mouse line). Scale bar: 100 µm. Boxed areas in A-F show interstitially localized X-gal-positive cells and are shown magnified below each panel (scale bar: 50 µm). (G-L) X-gal and Eosin staining of TA muscle sections 10 days following barium chloride injection, obtained from *pJ2-Cre:EGFP/+; R26^NZG/+^* lines #6 (G,H), #7 (I,J) and #11 (K,L) at >8 weeks of age (*n*=2 animals per mouse line). Scale bar: 100 µm. All X-gal-positive cells had centralized myonuclei. No X-gal signals were observed in *R26^NZG/+^* TA muscles (Fig. S5). Boxed areas in in G-L are shown magnified below each panel (scale bar: 50 µm).

Although the *DUX4* DMEs are known to be active in human and murine myogenic cells (Himeda et al., 2014), we did not see a strong X-gal signal in skeletal muscle when using whole-mount staining, which is consistent with rare expression of DUX4 in FSHD skeletal muscle (Jones et al., 2012; Tassin et al., 2013). However, cross-sections of TA muscles showed the presence of X-gal-positive cells in all three *pJ2-Cre:EGFP; R26^NZG/+^* transgenic lines (Fig. 6). While X-gal-positive cells were detected within myofibers (Fig. S4), they were also located outside of myofibers and within the interstitial space (Fig. 6). Interestingly, in all three *pJ2-Cre:EGFP; R26^NZG/+^* transgenic lines, X-gal-positive cells were often located near blood vessels in TA muscles (Fig. 6). Since DUX4 expression is causal for FSHD (Lemmers et al., 2010; Snider et al., 2010), and FSHD pathology is associated with active muscle regeneration (Snider et al., 2010), we investigated whether the *DUX4* regulatory elements are active during skeletal muscle regeneration. Skeletal muscle injury was induced in *pJ2-Cre:EGFP; R26^NZG/+^*mice by injecting barium chloride (BaCl_2_) into the TA muscle and then allowing regeneration to occur. At 10 days post injury, the centralized myonuclei of regenerating myofibers were found to be X-gal positive (Fig. 6G-L and Fig. S5).

**Fig. 7.**
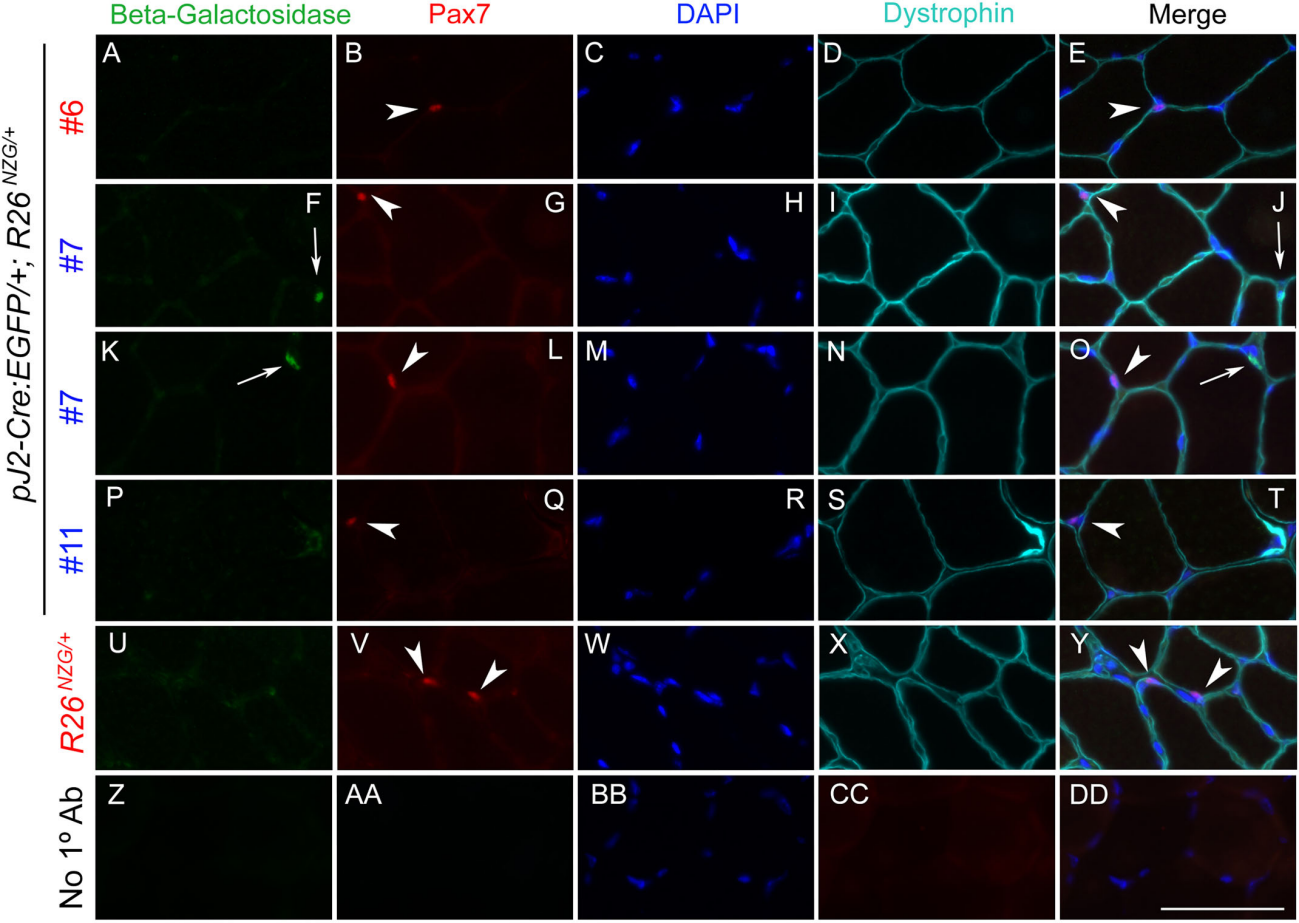
**The interstitially localized cells in which *DUX4* regulatory elements are active are not PAX7-positive muscle satellite cells.** (A-DD) Tibialis anterior (TA) muscle sections obtained from male (blue) or female (red) pJ2*-Cre:EGFP/+; R26^NZG/+^* mice (lines #6, #7 and #11) and *R26^NZG/+^* mice at 11 weeks of age were immunostained for β-gal (green; A,F,K,P,U), PAX7 (red; B,G,L,Q,V) or dystrophin (aqua; D,I,N,S,X). Line #7 has more panels because there were more PAX7 and β-gal-positive cells in sections from this line. Nuclei were stained with DAPI (blue; C,H,M,R,W,BB). Arrows show β-gal-positive nuclei; arrowheads show PAX7-positive nuclei. Scale bar: 50 µm. No 1° Ab, no primary antibody control.

These data suggested that DUX4-positive cell lineages are involved in muscle regeneration. Myogenic satellite cells are the major cell type contributing to muscle repair and regeneration (von Maltzahn et al., 2013; Wang and Rudnicki, 2011). The transcription factor PAX7 is expressed in quiescent and activated muscle satellite cells, and required for their function in adult skeletal muscle (Seale et al., 2000; von Maltzahn et al., 2013; Zammit et al., 2002). In addition, repression of PAX7 target genes correlates with FSHD disease status (Banerji, 2020). To determine if X-gal-positive lineages in skeletal muscle from adult *pJ2-Cre:EGFP; R26^NZG/+^* mice include satellite cells, muscle sections were co-immunostained for PAX7 and β-gal (Fig. 7; Fig. S6). As expected, cells expressing either PAX7 or β-gal were rare. Regardless, no cells showed staining for both. All detected PAX7-positive cells were negative for β-gal and, more importantly, all detected β-gal-positive cells were negative for PAX7. Thus, while we cannot exclude the possibility that a fraction of PAX7-positive cells are also X-gal positive and undetected in our analysis, our overall data indicate that the *DUX4* regulatory elements are not active in satellite cells but in a lineage of interstitial cells.

**Fig. 8.**
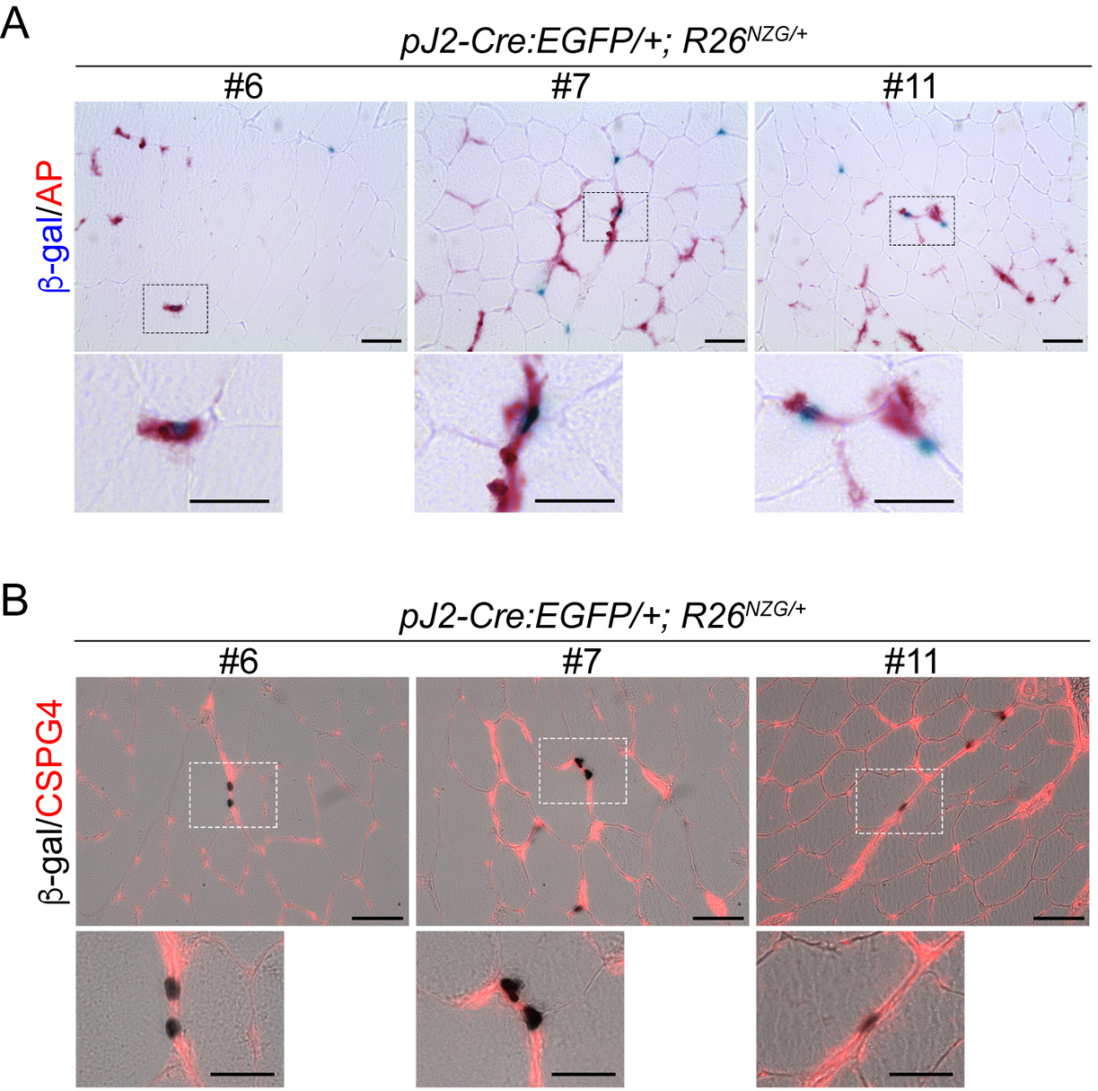
***DUX4* regulatory elements are active in the pericyte lineage. (**A,B) Images of normal tibialis anterior (TA) muscle sections obtained from pJ2*-Cre:EGFP/+; R26^NZG/+^* mice (lines #6, #7 and #11) at >8 weeks of age, stained for X-gal and alkaline phosphatase (AP) (A) or X-gal and the pericyte marker CSPG4 (B). Boxed areas are shown magnified below each panel. Scale bars: 50 μm (top panels), 25 µm (magnified bottom panels).

Pericytes, which are myogenic precursor cells distinct from myogenic satellite cells, are one of the cell types found in the small vessels of the skeletal muscle interstitial space (Dellavalle et al., 2007). To determine if the X-gal-positive interstitial cells in skeletal muscles of *pJ2-Cre:EGFP/+; R26^NZG/+^* mice are pericytes, skeletal muscle sections were stained for tissue nonspecific alkaline phosphatase (AP), a marker for pericytes and endothelial cells (Dellavalle et al., 2011). Indeed, some X-gal-positive cells in healthy muscles were positive for AP (Fig. 8A). Since AP is expressed in both pericytes and endothelial cells, we stained for the pericyte marker chondroitin sulfate proteoglycan 4 (CSPG4; also known as NG2) and found that some X-gal-positive cells are surrounded by CSPG4 (Fig. 8B). These data indicate that the *DUX4* regulatory regions were active at some point within the pericyte cell lineage.

## DISCUSSION

The goal of this study was to identify the tissues and cell lineages in which the human *DUX4* enhancer/promoter regulatory elements are active in mice. The scientific rationale for generating *pJ2-Cre:EGFP* transgenic mice was that, although the human *DUX4* gene is primate specific (Leidenroth et al., 2012; Leidenroth and Hewitt, 2010), many regulatory elements, pathways and transcription factors are conserved between mice and humans (Cheng et al., 2014; Diehl and Boyle, 2018). Interestingly, the *DUX* family genes show high conservation of expression and function with respect to zygotic genome activation across species (De Iaco et al., 2017; Hendrickson et al., 2017; Whiddon et al., 2017) and are encoded within D4Z4-like repeats, although the size of each RU and array varies (Clapp et al., 2007). Thus, the signals and trans-acting factors that regulate *DUX4* expression in humans with and without FSHD could be similarly conserved in mice, despite the fact that the DMEs do not appear to be present in the mouse genome. In fact, our initial work that identified and characterized the DMEs has shown that they are similarly active in both human and murine myogenic cells (Himeda et al., 2014), further validating this approach. In most non-FSHD individuals, *DUX4* is present in an extended D4Z4-repeat array, which is epigenetically silenced and thus, not expressed in somatic tissues, whereas in FSHD1, the D4Z4 array is contracted and *DUX4* is epigenetically de-repressed (de Greef et al., 2009; Himeda and Jones, 2019; Snider et al., 2010; van Deutekom et al., 1993; van Overveld et al., 2003; Wijmenga et al., 1992). Therefore, when crossed with *R26^NZG^* reporter mice, the *pJ2-Cre:EGFP* transgenic mice – which contain a single D4Z4 RU as found in severe FSHD1 – allowed us to visualize the activity of the *DUX4* regulatory elements *in vivo* under FSHD-like epigenetic conditions. Thus, they should be a useful tool for studying factors and stimuli that affect the regulation of *DUX4* expression in FSHD.

To some degree, *in vivo* regulation of *DUX4* within the context of a D4Z4-repeat array has previously been studied in the first published FSHD mouse models, the D4Z4-2.5 and D4Z4-12.5 mice, which differentially contain some of the human *DUX4* regulatory elements (Krom et al., 2013). The D4Z4-2.5 mouse transgene contains a 13.5-kb EcoRI-restriction fragment spanning the contracted chromosome 4q35 2.5RU D4Z4 array isolated from an FSHD patient, and including DME1 (but not DME2) and the FSHD-permissive 4A subtelomere, but lacking the downstream developmental non-coding exons 6 and 7, and the developmentally utilized PAS (Gabriëls et al., 1999; Snider et al., 2010). The D4Z4-12.5 mouse has significant additional centromere proximal sequence that contains the upstream *FRG1* and *FRG2* genes, and includes DME1, DME2 and the FSHD-permissive 4A subtelomere. While this mouse lacks the downstream developmental exons 6 and 7 and PAS, the healthy-sized 12.5 D4Z4 RU array causes epigenetic repression of the *DUX4* transgene (Krom et al., 2013). In comparison, the *pJ2-Cre:EGFP* mice used here have four key differences in transgene design. First, the pJ2-Cre:EGFP mouse has a single D4Z4 RU, albeit only the 1817 bp upstream of the *DUX4* open reading frame, with both DME1 and DME2. Thus, the genetic regulation is comparable to that of the non-FSHD 12.5-D4Z4 mouse, except that the pJ2-Cre:EGFP mouse is in the FSHD1 genetic state (1 D4Z4 RU) and, thus, not epigenetically repressed. Second, the Cre:EGFP fusion gene is in place of the *DUX4* gene and, therefore, Cre expression genetically marks cells and their lineages instead of potentially killing the cells (Kowaljow et al., 2007). Third, the Cre:EGFP reporter gene utilizes a β-globin PAS instead of the FSHD-permissive PAS sequence, while 2.5-D4Z4 and 12.5-D4Z4 mice lack the developmental PAS, which may or may not have consequences on developmental expression profiles. Last, the transgene integration sites are different for all mouse models, including chromosome 17 for the D4Z4-2.5 transgene and chromosome 2 for D4Z4-12.5 transgene (Krom et al., 2013). It should be noted that only single lines of both the D4Z4-2.5 and −12.5 mice were developed and analyzed, despite being generated by random insertion. Therefore, the effects of the integration site on the regulation of the D4Z4 transgene are not known. Overall, the pJ2-Cre:EGFP mice provide a unique opportunity to investigate developmental *DUX4* expression in the FSHD1-like state. Of note, the initial proof-of-concept analysis identified both murine testis and skeletal muscle tissue (Figs 2–5) as positive for *DUX4* expression, confirming what is known about *DUX4* expression in adult humans with and without FSHD (Jones et al., 2012; Snider et al., 2010; Yao et al., 2014).

### Human *DUX4* enhancer and promoter activity in mice

Comparing reporter expression among adult tissues of *pJ2-Cre:EGFP; R26^NZG^/+* mice, germ cells of the testis showed by far the highest and most uniform X-gal staining that was consistent between all three lines (Figs 4, 5). These results are similar to the abundant expression of *DUX4* in the testis of D4Z4-2.5 and D4Z4-12.5 mice (Krom et al., 2013), and humans (Snider et al., 2010). Moreover, *DUX4* expression in D4Z4-2.5 mice has been observed in cells near the periphery of seminiferous tubules, likely in spermatogonia and primary spermatocytes. The expression of *DUX4* in testis and in spermatogonia or spermatocytes has also been confirmed in human testis (Snider et al., 2010), indicating that the regulatory elements we used function similarly in murine and human testis.

With respect to somatic tissues, *pJ2-Cre:EGFP; R26^NZG^/+* adult mice showed some variable and line-specific tissue expression patterns outside of skeletal muscle (Fig. 4); however, double transgenic mice from all three lines exhibited positive, albeit very limited X-gal staining in skeletal muscles. Three independent-insertion lines of the transgene pJ2-Cre:EGFP led to X-gal staining in dorsal-anterior mesenchyme of limbs and a localized signal at the corner of the mouth, overlapping the general areas of clinical presentation in FSHD patient muscles. Within muscle sections, X-gal-positive staining was observed in the interstitial space, within myofibers and in the centralized myonuclei of regenerating myofibers. Since we did not observe GFP expression during ∼E10-E14.5 (Y.H., P.L.J., T.I.J., data not shown), the *DUX4* regulatory elements are likely active early in embryogenesis and not constitutively active over the course of development. Transgene integration effects are a concern in two of our lines (#6 and #11); however, line #7 displayed relatively consistent X-gal staining in the developing forelimbs, hindlimbs and face. The strong X-gal staining observed in these regions indicated that regulatory elements required for muscle-specific expression *in vivo* are present in the transgenic construct. However, the variable and limited spatial expression patterns throughout the developing musculature suggest that, in many places, the DMEs are not active. This is consistent with the poised state of these enhancers in cultured FSHD myocytes (Himeda et al., 2014). Non-transcriptional mechanisms, such as nonsense-mediated mRNA decay, may also play a role in limiting *DUX4-fl* expression (Feng et al., 2015). In FSHD, despite all of the cells sharing the same genetic defect, and similar epigenetic dysregulation of the pathogenic D4Z4 array and *DUX4* gene, it is extremely rare to find DUX4-positive cells in muscle biopsies from patients. While two studies have shown the presence of DUX4 protein in FSHD muscle biopsies by immunostaining (Beermann et al., 2022; Claus et al., 2023), the altered expression of DUX4 target genes generally serves as a surrogate of DUX4 activity (Tawil et al., 2024; Wong et al., 2024, 2020; Yao et al., 2014). Even cultured FSHD myotubes show only ∼1/200−1/1000 DUX4-positive myonuclei (Jones et al., 2012; Rickard et al., 2015; Tassin et al., 2013). Thus, the skeletal muscles of adult *pJ2-Cre:EGFP; R26^NZG^/+* mice exhibiting rare X-gal-positive nuclei, despite all cells in the tissue containing the same D4Z4 transgene and reporter gene, mimic the rare DUX4 protein expression found in FSHD muscles. While it is assumed that DUX4 expression within FSHD muscles occurs in myofibers, single-cell RNA-sequencing analysis will likely be required to correlate DUX4 target expression signatures with cell-specific signatures. The work presented here (Figs 7, 8; Fig. S6) suggests that PAX7-positive satellite cells are unlikely candidates to express DUX4, while pericytes may be a novel DUX4-expressing cell lineage in FSHD muscle.

With respect to spatiotemporal patterns of *DUX4* expression, the *pJ2-Cre:EGFP; R26^NZG^/+* mice fall somewhere between the D4Z4-2.5 and D4Z4-12.5 mouse models (Krom et al., 2013), which anchor opposite ends of the spectrum. With its short active array, the D4Z4-2.5 mouse displays almost ubiquitous *DUX4* expression (Krom et al., 2013). In contrast to this, D4Z4-12.5 mice contain an epigenetically repressed extended array, similar to the situation in non-FSHD individuals, with consequently rare and sporadic *DUX4* expression in only a few skeletal muscles, and virtually none in other somatic tissues (Krom et al., 2013).

Interestingly, D4Z4-2.5 mice contain DME1 but not DME2, suggesting that DME2 may serve to limit the cell types in which *DUX4* is expressed. Although it remains to be shown, we speculate that DME2 serves to both strengthen *DUX4* expression and limit the range of *DUX4* expressing tissues. One limitation of these transgenic models is that long-range chromatin associations that might influence normal expression of *DUX4-fl* (Petrov et al., 2008, 2006; Robin et al., 2015) are unlikely to take place in the context of the mouse genome. Nonetheless, even in this context, the DMEs appear to be active in cell lineages that give rise to both limb and different facial muscles, including those from the second branchial arch, which form the facial expression muscles that are commonly affected in FSHD. Consistent with this, the DMEs contain binding motifs for transcription factors (e.g. members of the TCF/LEF and SOX transcription factor families) downstream of signaling pathways that are critical for both limb and facial myogenesis (e.g. WNT signaling pathways) (Fitzsimons, 2011). The combinatorial complexity of transcriptional regulators that drives expression in different myogenic lineages has been well-documented but the interplay among factors binding the DMEs is still unexplored.

With regard to this, we acknowledge that the term ‘DUX4 myogenic enhancers’ may be misleading, considering that these regions have likely evolved to help activate *DUX4* expression at the cleavage stage of human embryogenesis and in the adult testis. As mentioned above, the DMEs contain binding motifs for powerful developmental transcription factors, and we postulate that these enhancers are most active at the stage when DUX4 is needed to drive human development. Additionally, the presence of binding sites for transcription factors important in skeletal muscle, including motifs for the myogenic regulatory factors, allows the DMEs to be active in myogenic lineages under circumstances when an active cognate promoter is present. In healthy muscle, the *DUX4* promoter is heavily repressed and the DMEs are not productively engaged but, in the FSHD state, the DMEs can engage with an active de-repressed *DUX4* promoter (Himeda et al., 2014). In skeletal myotubes, the DMEs display the marks of poised, rather than active, enhancers (Himeda et al., 2014); thus, they may respond to a combination of signals that occur together in only a fraction of cells, leading to the sporadic bursts of *DUX4* expression seen in FSHD muscle. It would be interesting to examine activity of the DMEs over the course of FSHD progression, to evaluate such models as the recently proposed rostro−caudal model (Padberg et al., 2023), but this would require a live-activity reporter rather than a lineage tracer.

Interestingly, DUX4 has been shown to activate expression of the histone variants H3.X and H3.Y (officially known as H3Y2 and H3Y1, respectively), which are then incorporated into the bodies of other DUX4 target genes, priming them for enhanced re-activation in response to a second burst of DUX4 expression (Resnick et al., 2019). Thus, early embryonic expression of DUX4 may establish epigenetic marks that contribute to the postnatal activation of target genes in the limbs and face, leading to disease progression. Unfortunately, the reporter lines in the present study cannot be used to investigate this theory, as the H3.X and H3.Y variants are primate specific.

### Pericytes have a DUX4-positive lineage

Skeletal muscle histology from all three lines of *pJ2-Cre:EGFP; R26^NZG^/+* adult mice showed X-gal-positive cells located both within myofibers and in the interstitial space near blood vessels. We detected the presence of X-gal-positive centralized myonuclei in regenerating fibers following muscle injury; however, immunostaining for PAX7 and β-gal failed to identify any X-gal-positive satellite cells (Fig. 7 and Fig. S6). While we cannot rule out the possibility that existence of a small population of X-gal-positive satellite cells was missed in our limited analysis, our results suggest that DUX4 was expressed in non-muscle cells that reside within the skeletal muscle and contribute to regeneration. Indeed, X-gal-positive interstitial cells were found to express pericyte cellular markers (Fig. 8). Pericytes, which arise from a distinctly different lineage than muscle satellite cells, also contribute to skeletal muscle regeneration (Dellavalle et al., 2011, 2007). Taken together, our findings suggest that *DUX4* regulatory elements are active in the pericyte lineage and that this lineage may have a previously unknown role in FSHD. Considering that DUX4 expression is detrimental to muscle development and often toxic to somatic cells (Bosnakovski et al., 2017, 2018; Kowaljow et al., 2007; Mitsuhashi et al., 2013; Rickard et al., 2015; Tassin et al., 2013; Wallace et al., 2011; Wuebbles et al., 2010; Yao et al., 2014), in FSHD, aberrant expression of DUX4 in the pericyte developmental lineage might adversely impact the pericyte cell population and/or function, potentially contributing to FSHD pathophysiology over time.

### New tools for FSHD research

Overall, these novel transgenic mouse models with human *DUX4* regulatory elements are potentially a powerful new tool for investigating the underlying causes of FSHD pathology. For example, this initial work (1) identified a blood vessel-associated cell lineage that, at some point in its developmental history, showed activated DUX4 expression and (2) implicated the pericyte cell lineage as a novel source of developmental DUX4 expression that could impact skeletal muscle formation, growth, repair and regeneration, thus potentially playing a role in FSHD pathology. In addition, the rare presence of X-gal-positive myonuclei in skeletal muscle recapitulates the rare mosaic DUX4 expression in FSHD skeletal muscle. As discussed earlier, the D4Z4 repeat in pJ2-Cre:EGFP mice mimics the situation in FSHD, where the 4q35 D4Z4 array is epigenetically dysregulated in all cells and cell types (de Greef et al., 2009; Jones et al., 2014); however, despite the loss of this repression, only a small fraction of FSHD skeletal muscle cells express DUX4 at any given time (Haynes et al., 2018; Jones et al., 2012). Interestingly, *DUX4* expression is significantly increased when cultured FSHD muscle cells are subjected to certain stresses, such as viral infection (C.L.H., P.L.J., T.I.J., unpublished observations), oxidative stress or DNA damage (Grow et al., 2021; Sasaki-Honda et al., 2018; Smith et al., 2023). One potential explanation is that DUX4 may be a stress and/or hormone-responsive gene (Jones et al., 2015; Teveroni et al., 2017) and FSHD pathology is induced by currently unknown signals that cause short-term bursts of DUX4 expression in increasing numbers of myofibers. DUX4 expressed in isolated nuclei is thought to spread to nearby nuclei within the myofiber, producing a gradient of expression (Tassin et al., 2013) and ultimately leading to the development of muscle pathology (Lim et al., 2020; Rickard et al., 2015). The *pJ2-Cre:EGFP; R26^NZG^* mouse model could be used to identify factors, conditions or stimuli that induce *DUX4* expression and, conversely, factors that prevent *DUX4* expression *in vivo*. Thus, our developmental models open up new *in vivo* discovery and therapeutic validation opportunities for understanding and treating FSHD.

## MATERIAL AND METHODS

### Animals

All animal procedures were approved by the University of Nevada, Reno IACUC (Protocol #0701). Euthanasia was performed using CO_2_ followed by cervical dislocation. pJ2-Cre:EGFP mice were generated by P.L.J. and T.I.J. at the University of Massachusetts Medical School transgenic mouse facility. ACTA1-Cre mice (strain #006149) (Miniou et al., 1999) and *R26^NZG^* mice (stock #012429) (Yamamoto et al., 2009) were purchased from The Jackson Laboratory. Genotyping primers for Cre recombinase are listed in Table S2. For the skeletal muscle injury procedure, 60−100 µl BaCl_2_ (1.2%) was injected into the right tibialis anterior (TA) muscle with 31G needle under isoflurane anesthesia.

### Transgene construction

The pJ2-Cre:EGFP transgene was generated by cleaving the pJ2 plasmid (Himeda et al., 2014) with FseI and AscI to remove the *DUX4* coding sequence. The replacement sequence was synthesized from FseI to the *DUX4* MAL start codon followed by the ATG and coding sequence for Cre:EGFP and the β-globin polyadenylation signal (PAS) termination cassette, based on the published pCAG-Cre:GFP sequence (Matsuda and Cepko, 2007), with an AscI restriction site added at the 3′ end. This FseI/AscI fragment was inserted into the similarly digested pJ2 vector to create pJ2-Cre:EGFP and validated by sequencing.

### Mapping transgene integration sites

For identification of transgene integration sites in pJ2-Cre:EGFP mice, viable frozen mouse spleen cells (pJ2-Cre:EGFP #6) and bone marrow (pJ2-Cre:EGFP #7 and #11) were used and processed by Cergentis (Utrecht, The Netherlands) using the targeted locus amplification (TLA) protocol (de Vree et al., 2014). Two primer sets to the transgene were designed and used in individual TLA amplifications (Table S2). PCR products were purified and the library prepared using the Illumina Nextera flex protocol, and sequenced on an Illumina sequencer. Reads were mapped using BWA-SW, version 0.7.15-r1140, settings bwasw-b7 (Li and Durbin, 2010). The sequencing reads were aligned to the transgene sequence and the mouse mm10 genome was used as the host reference genome sequence. The PCR primers for confirming the integration sites are listed in Table S2. PCR was performed using 100 ng of genomic DNA from each pJ2-Cre:EGFP mouse, GoTaq DNA polymerase, 200 µM dNTP, 200 nM each forward and reverse primer, with PCR program: one cycle at 94ºC for 3 min; 35 cycles at 94ºC for 20 s, at 60ºC for 20 s, at 72ºC for 35 s; one cycle at 72ºC for 2 min.

### X-gal staining

A Leica CM1950 cryostat was used for making 10 µm cross-sections from skeletal muscle and testis frozen in liquid nitrogen-cooled isopentane. Fixation was performed in a solution of 2% paraformaldehyde (PFA), 0.25% glutaraldehyde, and 0.05% NP-40 for 15 min when using a cryosection,1-2 h for embryos, and 1 h for adult tissues, followed by washing three times in 1× PBS. Samples were then immersed in X-gal solution (1 mg/ml X-gal, 5 mM potassium ferricyanide, 5 mM potassium ferrocyanide and 2 mM MgCl_2_) at 37°C for 45–120 min. Sperm smears derived from cauda epididymis were immersed in X-gal solution 37°C for 30 min without fixation. For X-gal plus eosin staining, after X-gal staining for 1 h, cross-sections were immersed in eosin solution for 10 s followed by 70-100% ethanol and xylene. For X-gal plus alkaline phosphatase (AP) staining, cross-sections were fixed with 4% PFA for 10 min and immersed in X-gal solution at 37°C for 1 h and then followed by PermaRed/AP (Diagnostic Biosystems, K049) for 10 min at room temperature.

For X-gal plus CSPG4 immunostaining, the cross-sections were first immersed in X-gal solution at 37°C for 1 h and then followed by immunostaining. Fixation was performed in 4% PFA for 10 min followed by treatment with 0.25% Triton X-100 for 15 min and blocking solution (5% normal goat serum and 0.01% Triton X-100) for 30 min. The primary anti-CSPG4 antibody (Millipore, AB5320: 1:200) was incubated at 4°C overnight and then secondary antibody (Alexa 594 goat anti rabbit IgG, Invitrogen, A11037: 1:400) at room temperature for 1 h.

### Immunofluorescence

The cross-sections were fixed in 4% PFA for 10 min followed by treatment with 100 mM glycine for 10 min, 0.25% Triton X-100 for 20 min and blocking with M.O.M. Immunodetection kit (Vector Laboratories) for 30 min. The primary antibodies against β-gal (ICL, #CGAL-45A, 1:200), PAX7 (DSHB, 1:5), dystrophin (Abcam, ab15277, 1:200) and MYH1 (DSHB, MF20, 1:5) were incubated at 4°C overnight. Then secondary antibodies (goat anti-chicken IgY Daylight 488 (Thermo Fisher Scientific, SA5-10070, 1:300), Alexa 594 donkey anti-mouse IgG (Jackson ImmunoResearch, 715-586-151, 1:300) and Alexa 647 donkey anti-rabbit (Jackson ImmunoResearch, 711-606-152, 1:300) were incubated at room temperature for 1 h. Prolong Gold Antifade Mountant with DAPI (Thermo Fisher Scientific) was used for staining nuclei.

### Imaging

For images of sperm and a cross-section of skeletal muscle and testis, a Leica DM 2000 LED microscope, DFC290 camera and LAS V4.12 software were used. For images of embryo and adult tissue, a ZEISS Stem 2000-C microscope, Qimaging MP3.3-RTV-CLR-10 camera and Capture suite software were used. For images of embryos at E14.5, two images were merged using Photoshop. For images including immunofluorescence, a LEICA DMi8 microscope, DFC365 FX camera and LAS X software were used.

## Supplementary Material

10.1242/dmm.052637_sup1 Supplementary information
