# Supplementary material for: Transgenic mouse models for investigating human DUX4 expression during development and its roles in FSHD pathophysiology
Source: Dis Model Mech. 2026 May 18;19(5):dmm052637. doi: 10.1242/dmm.052637 (PMC13225712; doi:10.1242/dmm.052637)
Supplement: Supplementary information [file dmm-19-052637-s1.pdf]

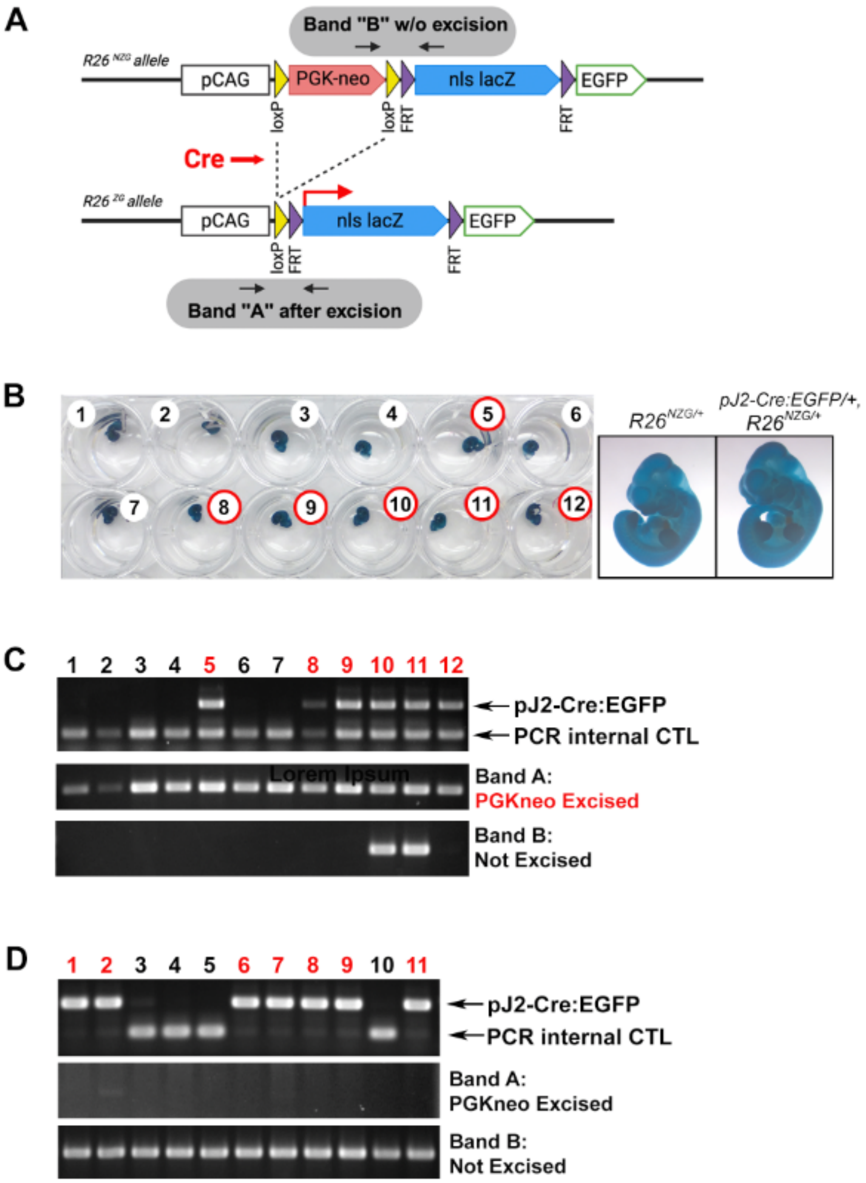

Fig. S1. Female *pJ2-Cre:EGFP/+* mice have maternal Cre expression during oogenesis, leading to excision of the floxed transgene in offspring in the absence of *DUX4* regulatory elements. A) Schematic of R26^{NZG} transgenes with and without Cre-mediated recombination. Genomic PCR band A shows Cre-mediated excision and genomic PCR band B shows the non-excised transgene. B, C) Heterozygous female *pJ2-Cre:EGFP/+* mice were crossed with homozygous *R26^{NZG}/R26^{NZG}* males and 12 embryos (E11.5) were analyzed by B) X-gal staining and C) genomic PCR using amniotic sac and umbilical cord genomic DNA. Double transgenic (red numbers) and *R26$^{NZG/+}$* heterozygotes (black numbers) all showed Cre-mediated excision (Band A) regardless of inheritance of the pJ2-Cre:EGFP transgene. D) Heterozygous male *pJ2-Cre:EGFP/+* mice were crossed with homozygous female *R26^{NZG}/R26^{NZG}* mice and 11 embryos (E13.5) were similarly analyzed by genomic PCR. Double transgenic embryos (red numbers) did not show any paternal Cre activity.

Disease Models & Mechanisms • Supplementary information

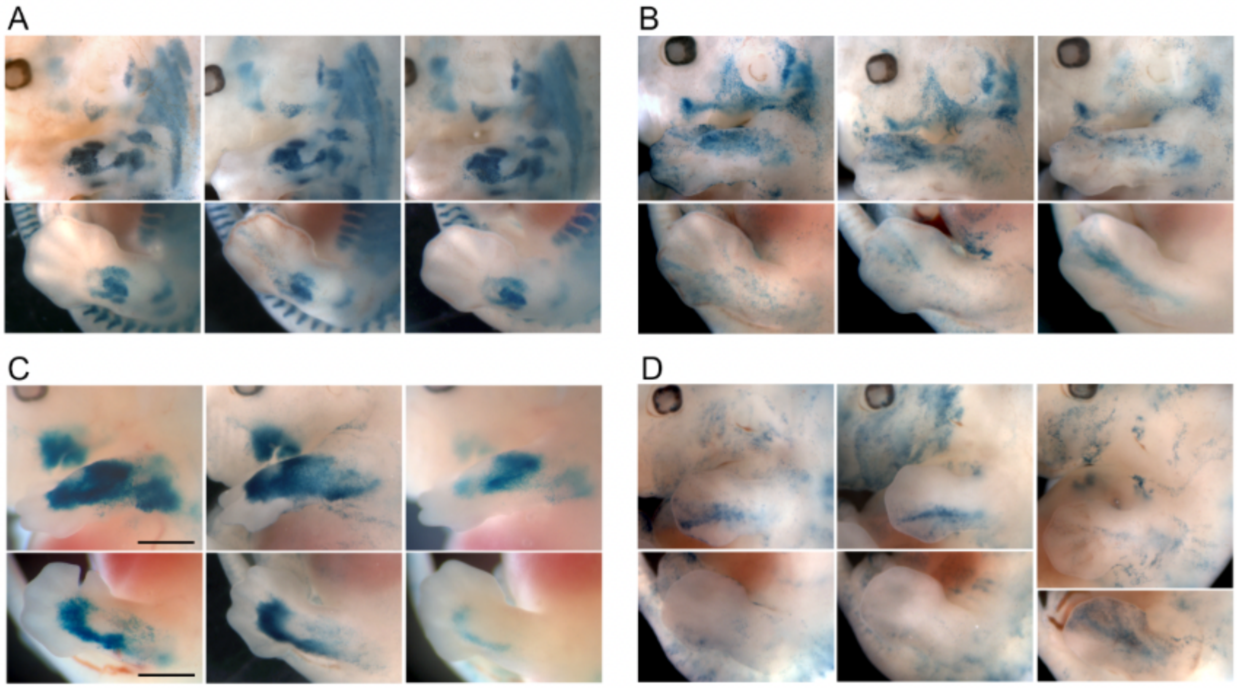

Fig. S2. Embryonic activity of *DUX4* regulatory elements among littermates. The close-up pictures of X-gal staining of three representative E13.5 embryos in the same litter of A) *ACTA1-cre/+, R26*$^{NZG/+}$ and B) line #6, C) line #7, and D) line #11 *pJ2-Cre:EGFP/+, R26*$^{NZG/+}$. X-gal staining in face and forelimb (top panels), and in hindlimb of the same embryos (bottom panels). Whole embryo images are also shown in Fig. 2 as follows: the second embryo in A is Fig. 2B, the first embryo in B is Fig. 2C, the first embryo in C is Fig. 2D, and the first embryo in D is Fig. 2E. Scale bars: 1 mm.

Disease Models & Mechanisms • Supplementary information

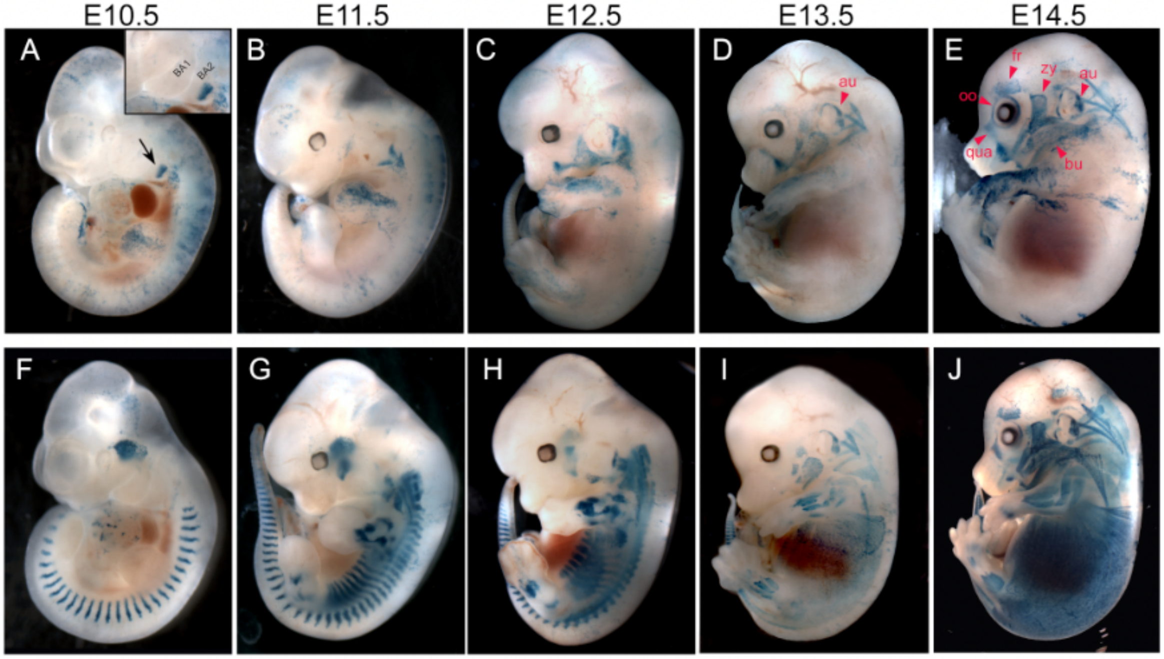

Fig. S3. *DUX4* regulatory elements in line #6 are active in the cell lineage of facial expression muscles during embryonic development. X-gal staining of line #6 *pJ2-Cre:EGFP/+; R26*$^{NZG/+}$ embryos (A-E), and *ACTA1-cre/+; R26*$^{NZG/+}$ embryos (F-J). A) At E10.5, line #6 shows the X-gal signal in the 2nd branchial arch (BA2, arrow). Closeup of BA2 signal is shown. B-E) Developmental activity of *DUX4* regulatory elements in line #6. X-gal staining suggests the positive cells in BA2 develop to form facial skeletal muscles located closer to the surface. Abbreviation of facial expression muscles in D and E are as follows: au, auricularis; bu, buccinator; fr, frontalis; oo, orbitalis oculi; qua, quadratus labii; zy, zygomaticus. Embryo images are also shown in Figure 2 as follows: 3C is Figure 2C, 3D is Figure 2H, 3E is Figure 2M, 3H is Figure 2B, 3I is Figure 2G, and 3J is Figure 2L.

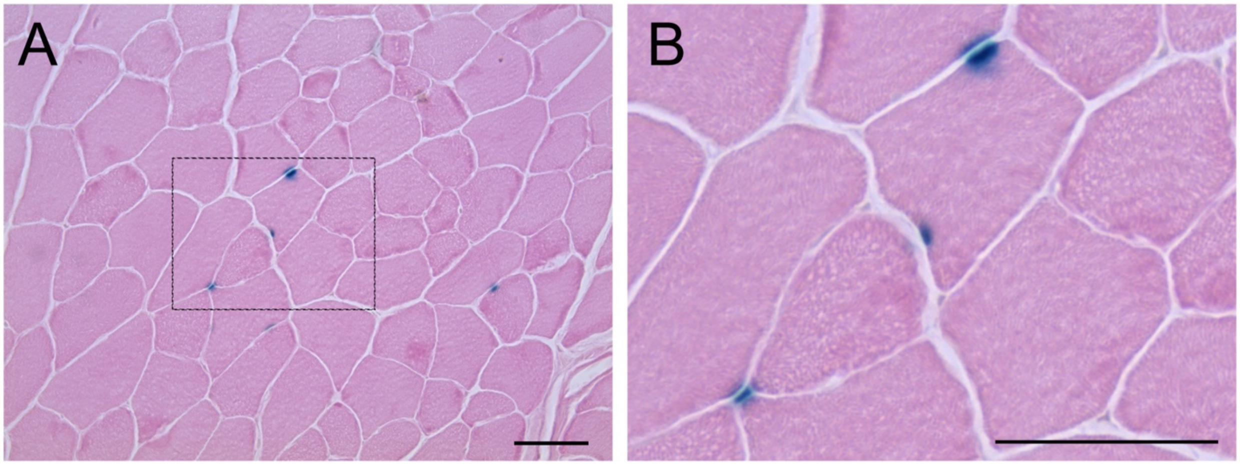

Fig. S4. Activity of *DUX4* regulatory elements in healthy skeletal muscle. A) X-gal staining of uninjured line #7 *pJ2-Cre:EGFP/+; R26*$^{NZG/+}$ mice at >8 weeks of age shows the signal in myonuclei. B) Enlargement of the rectangle in A. Scale bar: 50 µm.

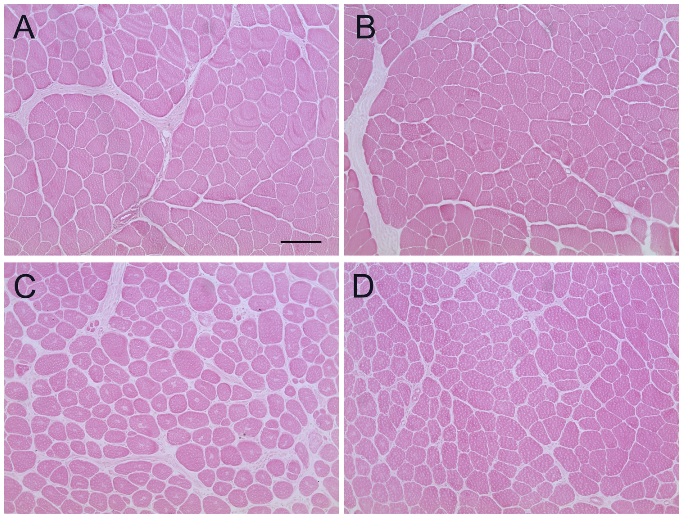

Fig. S5. R26$^{NZG/+}$ negative control for X-gal staining. X-gal and eosin staining of healthy (A and B) and injured TA muscles (C and D) TA muscles at 10 days after barium chloride injection. Scale bar: 100 µm.

Disease Models & Mechanisms • Supplementary information

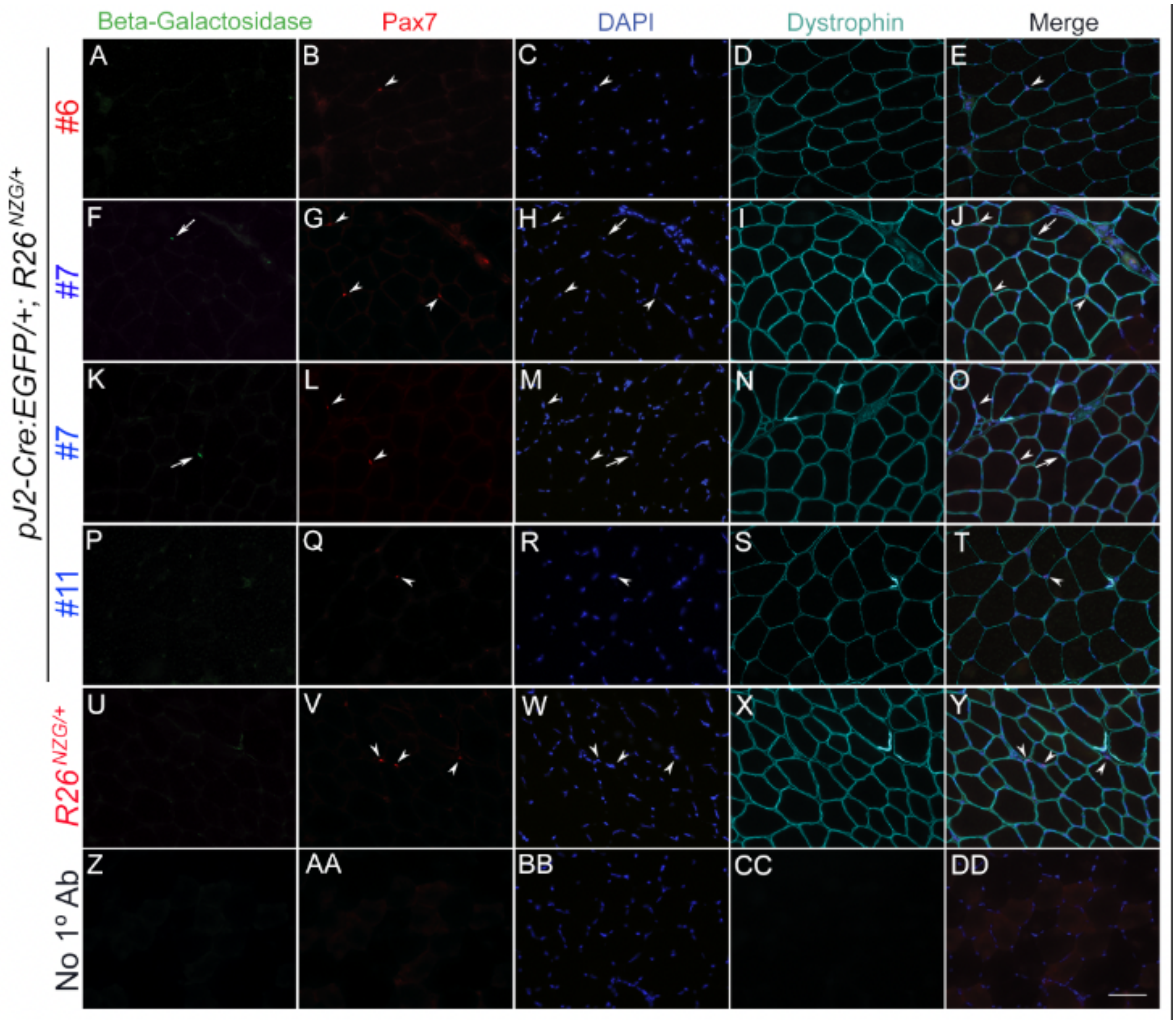

Fig. S6. The interstitially localized cells in which *DUX4* regulatory elements are active are not PAX7-positive muscle satellite cells. TA muscle sections from male (blue) and female (red) in indicated lines of *pJ2-Cre:EGFP/+; R26*$^{NZG/+}$ and *R26*$^{NZG/+}$ mice were immunostained for β-galactosidase (green, A, F, K, P, U), PAX7 (red, B, G, L, Q, V), and dystrophin (aqua, D, I, N, S, X), nuclei were stained with DAPI (blue, C, H, M, R, W, BB) to show nuclei. Z-DD) no primary antibody control. White arrows show β-galactosidase-positive nuclei and corresponding nuclei in DAPI panels; white arrowheads show PAX7-positive nuclei and corresponding nuclei in DAPI panels. These are the same immunofluorescent images used in Figure 7 but shown at smaller magnification. Scale bar: 50 μm.

Table S1. Consistency of X-gal signals observed in embryos from different litters

	pJ2-Cre:EGFP/+, R26$^{NZG/+}$ #6					*pJ2-Cre:EGFP/+, R26$^{NZG/+}$* #7				*pJ2-Cre:EGFP/+, R26$^{NZG/+}$* #11			
Stage	E12.5	E13.5	E13.5	E14.5	E14.5	E13.5	E13.5	E14.5	E14.5	E12.5	E13.5	E13.5	E14.5
Litter size	3	3	7	9	5	5	3	8	6	5	3	9	5
Face	3	3	7	9	5	5	3	8	6	5	3	9	5
Forelimb	3	3	7	9	5	5	3	8	6	5	3	9	5
Hindlimb	3	3	7	5	5	5	3	8	6	5	3	9	5
Trunk	3	3	4	5	5	0	3	4	1	5	3	9	5
Others*	0	0	0	0	2	0	0	0	0	5	3	7	4

Embryos at indicated stages were obtained from crosses of male *pJ2-Cre:EGFP/+* and female *R26$^{NZG/NZG}$* mice. Four to five litters were analyzed for β-galactosidase expression. The number of double transgenic embryos in each litter displaying X-gal signals in each anatomical region is shown. *X-gal signals in head and tail area. Note that X-gal staining of embryos tends to be variable at later stages (>E15) due to the larger size and higher density of the tissue that prevent efficient penetration of reagents; this likely affects the consistency of staining at E14.5.

Table S2. Oligonucleotide sequences.

Name	Sequence (5'-3')
Genotyping	
R47 #6 5' integration site F	gttggcaacttcagtgcaatc
R43 #6 5' integration site R	ccgagtcccggtcttttgtc
R44 #6 3' integration site F	cggaaggacatatgggaggg
R45 #6 3' integration site R	gtggggtgttgaaatctccc
R56 #7 5' integration site F	ggtggtcctggcttatttctc
R57 #7 5' integration site R	ggaatgtgtttgtgaagcacc
R58 #11 5' integration site F	gagccattgtggtgtttacct
R59 #11 5' integration site R	cagaacctgaagatgttcgcg
R54 #11 3' integration site F	ctctatgaacttccatgggacc
R55 #11 3' integration site R	cagaataccaatagcacaggc
Cre F	ttactgaccgtacaccaaaatttgcctgc
Cre R	cctggcagcgatcgctattttccatgagtg
Mapping analysis	
#6 Cre TLA F	ggagtttcaataccggagat
#6 Cre TLA R	attacgtatatcctggcagc
#7, #11 p13E-11 TLA F	cattcgaactcacaggca
#7, #11 p13E-11 TLA R	aactcccagtatctccttca
#6, #7, #11 GFP TLA F	caacagccacaacgtctata
#6, #7, #11 GFP TLA R	cgtccttgaagaagatggt
